# Saccades Matter: Reduced Need for Caloric Testing of Cochlear Implant Candidates by Joint Analysis of v-HIT Gain and Corrective Saccades

**DOI:** 10.3389/fneur.2021.676812

**Published:** 2021-06-28

**Authors:** Constanza Fuentealba Bassaletti, Babette F. van Esch, Jeroen J. Briaire, Peter Paul G. van Benthem, Erik F. Hensen, Johan H. M. Frijns

**Affiliations:** ^1^Department of Otorhinolaryngology and Head and Neck Surgery, Leiden University Medical Center, Leiden, Netherlands; ^2^Leiden Institute for Brain and Cognition, Leiden, Netherlands

**Keywords:** sensorineural hearing loss, cochlear implant, cochlear implantation, candidacy criteria, vestibular outcome, caloric test, v-HIT, vestibular areflexia

## Abstract

**Objectives:** Video head impulse test (v-HIT) is a quick, non-invasive and relatively cheap test to evaluate vestibular function compared to the caloric test. The latter is, however, needed to decide on the optimal side to perform cochlear implantation to avoid the risk on inducing a bilateral vestibular areflexia. This study evaluates the effectiveness of using the v-HIT to select cochlear implant (CI) candidates who require subsequent caloric testing before implantation, in that way reducing costs and patient burden at the same time.

**Study Design:** Retrospective study using clinical data from 83 adult CI-candidates, between 2015 and 2020 at the Leiden University Medical Center.

**Materials and Methods:** We used the v-HIT mean gain, MinGain_LR, the gain asymmetry (GA) and a newly defined parameter, MGS (Minimal Gain & Saccades) as different models to detect the group of patients that would need the caloric test to decide on the ear of implantation. The continuous model MGS was defined as the MinGain_LR, except for the cases with normal gain (both sides ≥0.8) where no corrective saccades were present. In the latter case MGS was defined to be 1.0 (the ideal gain value).

**Results:** The receiver operating characteristics curve showed a very good diagnostic accuracy with and area under the curve (AUC) of 0.81 for the model MGS. The v-HIT mean gain, the minimal gain and GA had a lower diagnostic capacity with an AUC of 0.70, 0.72, and 0.73, respectively. Using MGS, caloric testing could be avoided in 38 cases (a reduction of 46%), with a test sensitivity of 0.9 (i.e., missing 3 of 28 cases).

**Conclusions:** The newly developed model MGS balances the sensitivity and specificity of the v-HIT better than the more commonly evaluated parameters such as mean gain, MinGain_LR and GA. Therefore, taking the presence of corrective saccades into account in the evaluation of the v-HIT gain can considerably reduce the proportion of CI-candidates requiring additional caloric testing.

## Introduction

The cochlear implant (CI) presents an option of treatment for people with sensorineural hearing loss (SNHL) who benefit insufficiently from hearing aids. In many countries, including the Netherlands, only one CI per patient is reimbursed in the adult population. Unfortunately, there is currently no consensus on cochlear implantation criteria with respect to selecting the side of the primary implantation in bilateral SNHL (1–3).

In bilateral SNHL, the question whether the “worse” or the “better” hearing ear should be implanted is still under debate (3, 4). Some centers advise to implant the better hearing ear to obtain the best outcome from the implanted ear. This is based on the observed outcome when implantation was performed in ears with a shorter duration of deafness (4). Other centers, including ours, hesitate to give up hearing in the better hearing ear because of the risk of compromising a patient's communication abilities in case of a poor outcome of CI (5). The additional advantage of implanting the worst hearing ear is that there is more room for improvement with CI (6, 7).

Although it is questioned whether the vestibular state should play a significant role in the decision on the side of the implantation, West et al. showed that vestibulopathy was present in 25% of the pre-operative CI-candidates (8). This underscores the relevance of the vestibular evaluation as a part of the criteria for the selection of CI-candidates in order to prevent inducing bilateral vestibular areflexia. Therefore, it is often decided to select the better hearing ear for cochlear implantation if this ear has a vestibular areflexia and the only residual vestibular function is present in the contralateral ear.

Caloric testing is the gold standard to distinguish between vestibular areflexia and hyporeflexia (9), using a non-physiological stimulus but allowing for an ear-by-ear assessment, which is relevant in the context of the choice of CI side. However, it is relatively time consuming and places a considerable burden on the patients. In contrast, the video head impulse test (v-HIT), a non-invasive test to evaluate vestibular function, uses a physiological stimulus (i.e., head movements) and is relatively quick, cheap and less bothersome to patients compared to the caloric test. Classically, the vestibular-ocular reflex (VOR) gain is the main parameter to consider in order to classify vestibular dysfunction (9–12), and some researchers have suggested that the parameter of VOR asymmetry can be correlated with the canal paresis score (9). However, several studies have advocated the use of corrective saccades for this purpose, considering this phenomenon an indicator of a vestibular lesion (13–15). Therefore, one can argue that it is important to consider and analyze the previously mentioned v-HIT parameters to correctly classify a semicircular canal (SCC) dysfunction. This study evaluates the effectiveness of using the v-HIT to reduce costs and patient burden by selecting CI-candidates who do not require caloric testing before implantation.

## Materials and Methods

This is a retrospective cohort study comprising a complete review of CI data at Leiden University Medical Center (LUMC). We have examined the records of all 354 adult CI-recipients (age at implantation >18 yrs), implanted between 2015 and 2020. Only patients with a complete pre-operative caloric test and v-HIT results were included. Exclusion criteria were bilateral implantation, incomplete or unreliable caloric test and v-HIT results. The vestibular evaluation with the v-HIT was introduced at the LUMC in 2015 and has been increasingly used. Up till recently, however, it was not the standard of care for all CI-candidates. This is one of the reasons for the final inclusion of 83 patients.

### Subjects

The current study includes 83 patients (31 female, 37%), between 18 and 89 years of age at the time of the implantation (mean 60 yrs, SD (standard deviation) 13 yrs). The duration of deafness varied between 1 and 70 years (mean 20;02 yrs, SD 18;07 yrs). Bilateral SNHL was the diagnosis for 82 patients, and one patient had bilateral severe mixed hearing loss. Sixty-five patients had post-lingual deafness on the right ear, and sixty-six on the left ear. There were five patients with missing data on this matter. Data on the etiology of the hearing loss are summarized in Table 1. The CI was implanted in 42 candidates on the right side, and in 41 candidates on the left side. During the intake, all patients were asked whether they experienced vestibular symptoms. Forty-three patients had vestibular complaints, viz. imbalance (26.5%), dizziness (15.7%), vertigo (14.5%), imbalance in the dark (10.8%), oscillopsia (7.2%), vomiting (2.4%), falls (2.4%), light-headedness (2.4%). The other 40 patients did not exhibit vestibular symptoms.

**Table 1:** Etiology of hearing loss per ear.

**Etiology**	**Ear**	**Frequency (%)**
Idiopathic acquired	Left	65 (78.3)
	Right	61 (73.5)
Idiopathic congenital	Left	9 (10.8)
	Right	9 (10.8)
Ménière's disease	Left	0
	Right	5 (6)
Otosclerosis	Left	3 (3.6)
	Right	2 (2.4)
Rubella	Left	1 (1.2)
	Right	1 (1.2)
Birth asphyxia	Left	1 (1.2)
	Right	1 (1.2)
Usher syndrome	Left	1 (1.2)
	Right	1 (1.2)
Meningitis	Left	1 (1.2)
	Right	1 (1.2)
Premature birth	Left	1 (1.2)
	Right	1 (1.2)
DFN8	Left	1 (1.2)
	Right	1 (1.2)
Total	Left	83 (100)
	Right	83 (100)

### Caloric Test

The bithermal caloric testing, using cool and warm water at 30 and 44°C, respectively, was performed to provoke vestibular responses in both ears. The patient was in supine position with its head inclined at 30 degrees to the horizontal to bring the horizontal semicircular canal into the vertical plane. The eye movements were recorded with VNG system (Vestlab 7.0®, Otometrics, Germany). The caloric responses were measured in terms of the maximum slow-phase velocity (SPV) of the nystagmus in degrees per second. The canal paresis (CP) or unilateral weakness (UW) and directional preponderance (DP) were quantified according to the Jongkees formula in percentages (16). Caloric test results were considered abnormal if the unilateral weakness was ≥22%, the directional preponderance was ≥25% or the SPV was below 15°/s for each ear (9, 17). Vestibular areflexia was defined as a complete absence of caloric responses. The bilateral vestibulopathy was determined by a SPV below 6°/s in all four traces (warm right, warm left, cold right, and cold left). The outcomes were carefully reviewed and analyzed by three specialists within LUMC. A group of 28 patients was identified in which the caloric test results played a decisive role in selecting the optimal side for cochlear implantation.

### Video Head Impulse Test

The video head impulse test (v-HIT) of horizontal canal function was measured using the commercial video oculography system (ICS Impulse System, GN Otometrics, Denmark). During the test, the patients wore goggles with a built-in video camera that recorded real time eye movements. Before starting the test, a calibration was performed to ensure accurate recordings. Patients were tested while sitting upright in a lighted room with an eye level target at a minimum of 1 meter in front of them. They were asked to stare at the fixed target and minimize blinking, while the evaluator performed sharp and fast head rotations, delivered randomly to left and right. Horizontal v-HIT results were deemed acceptable when the peak head velocity reached 150–200°/s. The corrective saccades were traced as a delayed eye movement during (covert saccades) or after (overt saccades) the head movement. The constant presence of covert or overt catch-up saccades was considered as indicator of VOR abnormality. It turned out that 23 out of the 28 patients requiring a caloric test were in the group with corrective saccades. The VOR gain was calculated by the software as the ratio of peak slow phase eye velocity to peak head velocity (18, 19). In line with the literature, we defined a cut-off value of 0.8 for the v-HIT gain indicating an abnormal horizontal VOR (9, 20, 21).

**Figure 1:**
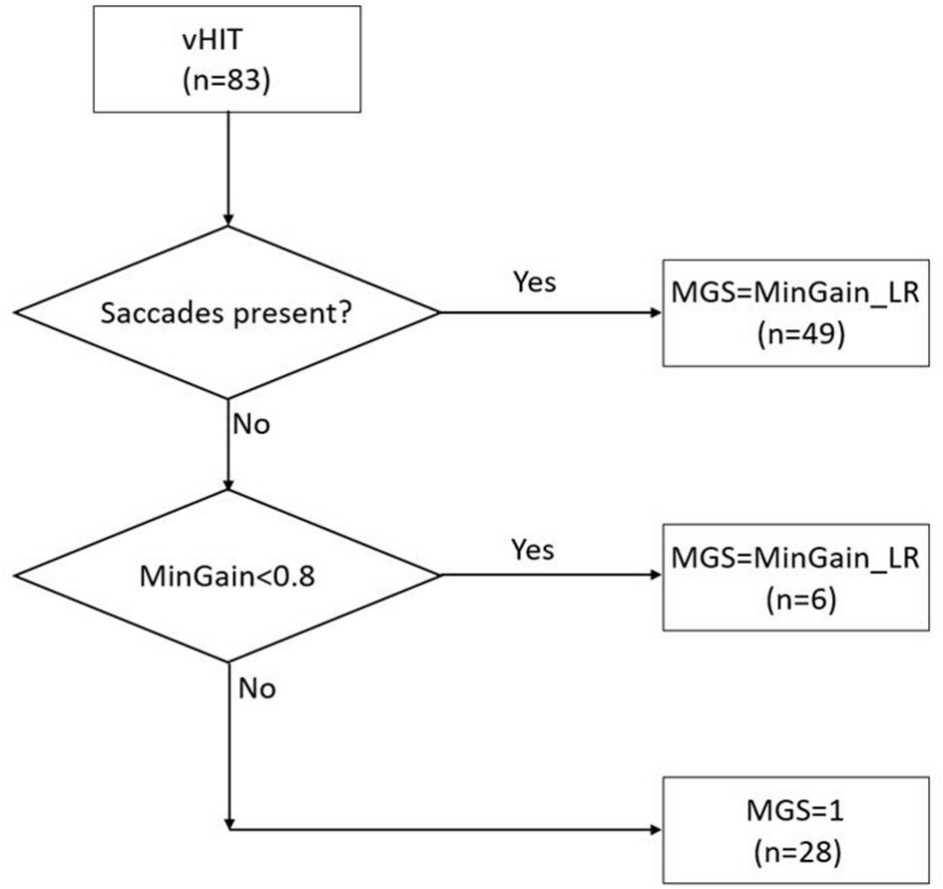
The MGS (minimal gain & saccades) value is calculated based on the combination of the presence of saccades in the v-HIT and MinGain_LR, the lower of the two values of the v-HIT gain to the left and right. The numbers between brackets indicate the number of patients in the various categories in the present study sample.

In order to be able to analyze the data with receiver operating characteristics (ROC) curves (see section **Statistical Analysis**), the v-HIT outcomes were used as a continuous variable. We used the v-HIT mean gain, minimal gain of both ears and the gain asymmetry (GA) as different models to select the group of patients that would require the caloric test to decide the side of implantation. In addition, we wanted to make use of the abovementioned observation that the vast majority of CI-candidates requiring caloric testing exhibit corrective saccades, and to combine it with the intuitive parameter of at least unilateral low gain, i.e., minimal gain of left and right ear (MinGain_LR). The presence of saccades was checked visually by two authors (CFB and BFE) independently, disagreement was resolved with a consensus discussion. In fact, we had three groups of patients: (a) 49 cases with corrective saccades (irrespective of the gain), (b) 28 cases with normal gain (≥0.8) and no saccades, and (c) six cases with abnormal gain (<0.8) without saccades. Therefore, we introduced the continuous model MGS (Minimal Gain & Saccades). MGS is defined as MinGain_LR, the lower of the two values of the v-HIT gain to the left and right, except for the cases with normal gain (both sides ≥0.8) where no corrective saccades were present (group b). In the latter case, MGS was defined to be 1.0 (the ideal gain value). This is further illustrated in Figure 1.

### Statistical Analysis

We performed descriptive statistics for categorical variables, including sex, age, hearing loss etiology by means of the IBM SPSS Statistics v.25. Means and SDs were calculated for age and duration of deafness.

First, we evaluated to what extent the side with vestibulopathy or areflexia found with the caloric test result corresponded with an abnormal horizontal angular VOR as found with the v-HIT. Differences between groups were assessed by means of cross-tabulation and analyzed using the Chi-square test. A *p*-value <0.05 was considered significant. Positive predictive value (PPV), negative predictive value (NPV), sensitivity and specificity and their 95% confidence interval (CoIn) were calculated with the online software of MedCalc (available at www.medcalc.org).

More importantly, ROC curves were constructed to analyze the sensitivity, specificity and area under the curve (AUC) values for the various v-HIT parameters. An ROC curve is a graphical plot that is commonly used to analyze a test's ability to discriminate between a subject with and without a disease (22). In this study such ROC curves were used to evaluate to which extent the various v-HIT parameters (also called models) can be used to determine whether a CI-candidate needs additional caloric testing to decide on the side of implantation. The sensitivity in this curve (vertical axis) indicates the proportion of candidates requiring calorics that is detected by the test (“true positive rate”). The horizontal axis denotes the “false positive rate,” also known as “1-specificity” shows the fraction of cases that would undergo caloric testing despite the fact that they don't need it. The v-HIT mean gain, MinGain_LR, the GA and the MGS were used as continuous variables in this analysis, for which the optimal cut-off points can be determined. The analysis also included the construction of the curve showing the trade-off between increasing the sensitivity and the number of CI-candidates who need additional caloric testing.

## Results

Caloric test showed abnormal results in 28 out of 83 patients (34%). The mean UW was 26% (SD 25%) and the mean DP was 21% (SD 23%). Complete bilateral areflexia was found in 4 (14%) of the patients and asymmetrical hypofunction in 24 patients (86%). Twelve patients had hypofunction in both the left and the right side. All but one patient (who had a left gain of 0.76 and right gain of 0.75) with bilateral low gain in the v-HIT also had bilateral areflexia in the caloric test.

The v-HIT gain was abnormal in 20 (24%) out of 83 patients. The v-HIT results showed a mean gain of 0.87 (SD 0.16; Range 0.11–1.15) to the left and 0.94 (SD 0.22; Range 0.05–1.36) to the right. In total 20 patients had a VOR gain below 0.8, of which six did not exhibit corrective saccades. Of the remaining patients with a VOR gain below 0.8, eight had just overt saccades and six had both overt and covert saccades. Eight patients had a normal VOR gain and presence of corrective saccades.

Data of caloric testing and v-HIT results per patient is represented in Supplementary Digital Content 1.

Table 2 directly compares the v-HIT outcomes with the caloric test results in a classical way. As explained in the Methods section, caloric test results were considered abnormal if the unilateral weakness was ≥22%, the directional preponderance was ≥25% or the SPV was below 15°/s for at least one ear, while a v-HIT was considered to show vestibular dysfunction if the mean VOR gain was <0.8. It turned out that the v-HIT, used in this way to predict an abnormal caloric test, had a positive predictive value (PPV) of 65% (95% CoIn: 46–81%), a negative predictive value (NPV) of 76% (95% CoIn: 69–82%), a sensitivity of 46 % (95% CoIn: 28–66%) and a specificity of 87% (95% CoIn: 76–95%).

**Table 2:** Results of the caloric testing and the v-HIT with an horizontal VOR gain cut-off value of <0.8.

**v-HIT**	Caloric test^*^		**Total**
	**Abnormal**	**Normal**	
Gain <0.8	13	7	**20**
Gain ≥ 0.8	15	48	**63**
**Total**	**28**	**55**	**83**

*Sensitivity was 46% (95% CoIn: 28–66%), specificity 87 % (95% CoIn: 76–95%), positive predictive value (PPV) 65% (95% CoIn: 46–81%), negative predictive value (NPV) 76% (95% CoIn: 69–82%).*

* *For abnormal caloric test, cut-off values were a UW ≥22%, DP ≥25% and/or a SPV <15°/s for each ear*.

**Figure 2:**
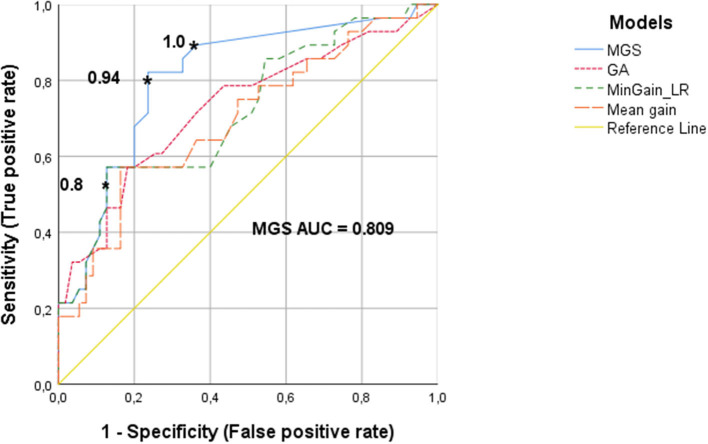
ROC curves of the v-HIT for determining the need for an additional caloric test, using the parameters MGS (minimal gain & saccades), GA (gain asymmetry), MinGain_LR and mean gain. The asterisks show the cut-off values for the MGS parameter. The cut-off point of 0.8 resulted in 87% specificity and 49% sensitivity. The value 0.94 has a specificity of 76 and 80% sensitivity. The cut-off value of 1 has a specificity of 62% with a sensitivity of 90%.

Figure 2 shows the ROC curves, which quantify the trade-off between true positives and false positives when deciding whether a CI-candidate will need additional caloric testing on the basis of v-HIT outcomes. The cut-off values of v-HIT mean gain, MinGain_LR, the GA and the MGS were continuously varied as described in the Methods section.

The ROC curve for the v-HIT mean gain had an AUC of 0.70 (95% CoIn 0.57–0.82), which means that for this commonly used variable the model has a poor-to-moderate diagnostic capacity. For MinGain_LR, the AUC was 0.72 (95% CoIn 0.60–0.84), which represents a good diagnostic capacity. If we select the common cut-off point of 0.8, the sensitivity is 49% and the specificity is 87%. When the sensitivity is increased to 80% (cut-off point at 0.93), the specificity decreases to 47%. In case of selecting a sensitivity of 90% (cut-off point at 0.99), the specificity is lowered to 27%. The percentage of GA between the left and right ear had an AUC of 0.73 (95% CoIn 0.61–0.85), also representing a good diagnostic capacity. A sensitivity of 80% was associated with a specificity of 48%, and reached for the cut-off point GA = 7.7. Ninety percent sensitivity was reached for the cut-off point of 4.5 for GA, with a specificity of just 25%. The newly designed parameter MGS, had an AUC of 0.81 (95% CoIn 0.71–0.91), representing a very good diagnostic accuracy for identifying subjects needing caloric testing. When we selected a cut-off point of 0.8 for MGS, this resulted in a 87% specificity and we found a sensitivity of 49%. For a sensitivity of 80%, the cut-off point for MGS is 0.94, with a specificity of 76%. If we want to improve the sensitivity up to 90% (i.e., missing just 10% of cases requiring caloric testing) the cut-off value of MGS is 1, and the specificity decreases to 62%.

**Figure 3:**
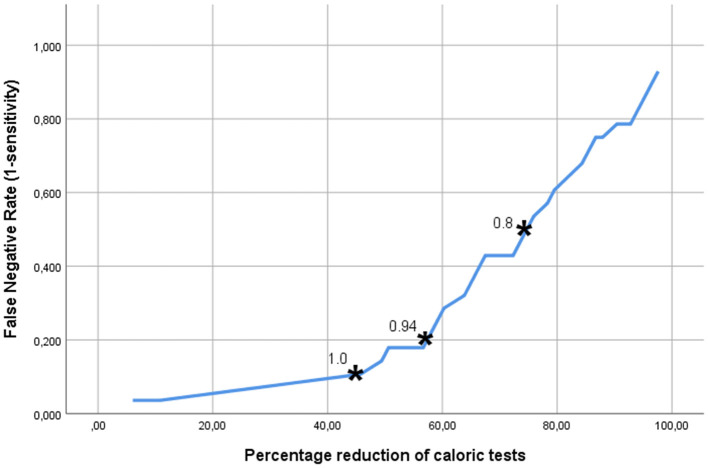
The trade-off between the accepted fraction of CI-candidates requiring caloric testing, but not undergoing it, and the reduction in the number of the caloric tests, which can be achieved if the MGS (minimal gain & saccades) is used as discriminating parameter. The asterisks indicate the points on the curve representing the MGS cut-off points 0.8, 0.9, and 1.0. The latter cut-off value, associated with a sensitivity of 90% resulted in a reduction of the number of patients in need of caloric testing by 46%.

Figure 3 shows the trade-off between the desired sensitivity and the percentage reduction of patients undergoing caloric tests (horizontal axis) if MGS, the best test parameter, is used. The vertical axis denotes the “false negative rate” (1-sensitivity), i.e., the fraction of CI-candidates requiring caloric testing, but not undergoing it. The asterisks indicate the points on the curve, representing the abovementioned cut-off points of MGS (0.8, 0.94, and 1.0). From this analysis it became clear that the latter cut-off value, associated with a sensitivity of 90% resulted in a reduction of the number of patients needing to undergo caloric testing by 46%.

## Discussion

### Key Findings

In this retrospective study, our aim was to evaluate the effectiveness of the v-HIT to select CI-candidates who require caloric testing before the implantation in order to use this information as part of the selection criteria, thereby avoiding the induction of bilateral vestibular areflexia. The v-HIT conveys different parameters that reflect the VOR function. Usually, the gain is used to identify the lesion side and magnitude of dysfunction (10–12). In our study we analyzed the mean gain, MinGain_LR, the GA and the MGS (a novel combination of gain and/or presence of corrective saccades) to identify the cases with abnormal caloric function. The AUC of 0.81 found for the MGS parameter, indicates a very good accuracy of this model. Further analysis (Figure 3) indicated that one can dispense of almost half of the caloric tests with false negative rate of 10% (i.e., missing 10% of the CI-candidates that need caloric testing) if the vestibular assessment is started with a v-HIT evaluation based on the MGS. The ROC curves analysis for the v-HIT mean gain, MinGain_LR and GA showed a lower AUC. Therefore, the MGS model was identified as the one that better balances the true positive and the false positive rate for predicting the necessity of a further caloric test in CI-candidates.

It is possible to observe and analyze different parameters of the v-HIT, however the average gain of the VOR is one of the variables that is classically chosen to decide the vestibular hypofunction side (10–12). In our data set we could confirm other authors' findings (17, 21, 23) with respect to sensitivity and specificity of the v-HIT gain compared to the caloric test as the golden standard: When using the cut-off value of v-HIT gain <0.8 to classify vestibular hypofunction, we found a PPV of 65%, a NPV of 76%, a sensitivity of 46% and a specificity of 87%, which is in line with the literature.

### Comparison With Other Studies

To our knowledge this is the first study that included the presence of corrective saccades in the analysis to determine if further caloric testing is necessary in CI-candidates. Such corrective saccades will occur when the VOR is insufficient to keep the gaze on the target, i.e., move the eyes at the same velocity of the head movement. Thus, the brainstem will compensate by generating corrective saccades to adjust the eyes back to the earth-fixed target (24, 25). Their presence could indicate an abnormality of the VOR or that vestibular compensation is taking place, as demonstrated in previous studies, which underscore the relevance of considering the corrective saccade as a variable that denotes SCC dysfunction, besides the gain value (13–15, 26, 27).

Janky et al. characterized saccades in a control group and then compared these data to subjects with vestibular loss (14). Their analysis showed that a combined gain value <0.78 with a corrective saccade frequency >81.89% resulted in a 90% specificity and 78.8% sensitivity, with an overall correct classification rate of 84.6%, compared with the v-HIT gain value alone. They suggested that the presence of repeatable saccades could indicate a VOR deficit, regardless the gain value, indicating v-HIT abnormality. In our study, the MGS model was obtained with a formula that included the presence of corrective saccades, even if the gain was normal (>0.8), see Figure 1. With 87% our specificity was similar to Janky et al., but the sensitivity was 49% with a cut-off value of 0.8 in the ROC curve. This difference can be explained by the methodology used. In their study, Janky et al. analyzed the first corrective saccade based on his frequency, peak velocity and latency, where in our sample with CI-candidates we classified the corrective saccades as present or absent. Also, it is relevant to mention that Janky et al. studied the value of corrective saccades in a group of patients to diagnose vestibular loss, which is different from our aim that was to use the v-HIT parameters to specifically identify CI-candidates who need additional caloric testing.

Other studies reported the presence of corrective saccades and normal VOR gain values in subjects after CI surgery (13, 28). The authors postulated that the corrective saccades may represent a partial dysfunction of the VOR and that the gain by itself might not reflect all the physiologic changes after a CI surgery, which affects the vestibular function.

In the field of otoneurology, several studies have been comparing the caloric test and the v-HIT performance to show their predictive values as a diagnostic tool. Moreover, many researchers and clinicians have wondered whether it is necessary to use both tests. However, we must remember that the v-HIT evaluates the VOR at a high frequency of stimulation, >5 Hz, during a physiological head movement, while the caloric test evaluates the vestibular system at a low frequency, 0.003 Hz, during a non-physiological ear irrigation. As a result, both tests provide complementary information (9).

We noticed a high specificity and moderate sensitivity of the v-HIT gain using the caloric testing as a reference. Similar outcomes were presented in previous studies when comparing both vestibular tests (17, 21, 23, 29, 30). However, the study of Aalling et al. (29) showed a higher PPV of 90% compared with our PPV of 65%. This could be explained by the fact that their study evaluated all 6 semicircular canals, whereas we evaluated only the 2 horizontal semicircular canals, making their assessment more accurate. The other studies did not mention PPV or NPV (17, 21, 23, 30). Beynon et al. (17) made a correlation between the severity of the caloric hyporeflexia and a higher true positive rate; of those patients with complete canal areflexia, 87% (21 out of 24) had a positive v-HIT result. That is also shown in our study, but to a lower scale (75%; 3 out of 4 patients).

Other studies (31, 32) reported different sensitivity and specificity values. This could be explained because they used a different cut-off point to classify the caloric hypofunction, viz., an absolute value of UW 25% (31). In Bartolomeo et al. (32) study, the higher sensitivity of 100% in the v-HIT was found when the caloric hypofunction was ≥62.5%. Their mean caloric vestibular deficit in a vestibular neuritis population was 78.7 ± 21.24%, which is considerably higher than our population of CI-candidates (26 ± 25%). This fact might explain the substantial difference in sensitivity. In our study, the presence of a value of ≥22% UW, ≥25% DP or a <15°/s SPV, classifies the result of a caloric test as abnormal. In that respect, our classification is more refined than the aforementioned studies in identifying vestibular dysfunctions.

A previous study on v-HIT normalization with 50 healthy subjects found that 100% of the subjects had a GA below 8% (20). However, this is not the value being used in the clinical practice. Clinicians usually consider GA to be normal between 0 and 13% (9). Although the use of the GA is not universally used to classify a patient with vestibular dysfunction, we have decided to include this parameter due to his comparability with the canal paresis score from the caloric test (9).

### Strengths of the Study

Based on our results, corrective saccades (as taken into account with the MGS parameter) have added value for interpreting the VOR in CI-candidates. The saccades can show subtleties in the VOR function, providing objective evidence of changes in SCC function that sometimes the v-HIT gain alone will not explain completely. It turned out that using just the gain value as a main parameter could guide us into an overestimation of the vestibular function of the subject. Moreover, the present study showed that by using the MGS to include the presence of corrective saccades in the analysis, the v-HIT -contrary to expectations based upon classical parameters- is effective to select CI-candidates who will require caloric testing before surgery, reducing patients burden and costs.

### Limitations of the Study

It is important to be mindful of the limitations of this study in order to interpret study results. This a retrospective cohort study based on the information retrieved from the files of CI-candidates, and not patients who specifically complained about vestibular symptoms like in most studies. Although CI-candidates were exactly the population we had in mind for the research question, one has to consider the presence of selection bias when trying to generalize the outcomes, e.g., to a specific population with vestibular dysfunction. In this context it is relevant to mention that 33.7% of the CI-candidates had a vestibular hypofunction as measured by means of the caloric test.

As explained in the introduction, the vestibular evaluation with the v-HIT was not a standard of care for the population of CI-candidates in our center until recently. As a result, only 23% (83 out of 354) of the CI-candidates in the period 2015–2020 had a complete vestibular assessment, including caloric testing and v-HIT, allowing them to be included in this study.

Another limitation to take into account is that the MGS does not reflect a per ear analysis. However, the test characteristics for v-HIT gain per ear turned out to be even poorer when identifying the patients who need calorics. Therefore, these scores cannot be used in clinical practice to directly diagnose the best side of implantation since the side with the most prominent vestibular loss is not identified. Thus, we could not predict the side of the hypofunction as a caloric test could do, but the data allowed to decide whether an additional caloric test is warranted in a particular CI-candidate.

### Clinical Applications

Although the classic analysis considers the v-HIT gain value as the main VOR status parameter, we strongly advise to also consider the corrective saccades as an additional parameter when classifying a vestibular dysfunction. Using the v-HIT (with MGS as the main parameter) at the beginning of the vestibular evaluation of CI-candidates, and more importantly before the caloric test, could help us to eliminate almost 50% of the caloric assessments, by finding the cases that do not need caloric testing to identify the side with the vestibular hypofunction. As a result, starting with the v-HIT could optimize both the evaluation time per patient (31) and the invasiveness of the diagnostic trajectory. However, it is necessary to have a group of experienced professionals who are able to correctly identify the presence of corrective saccades, despite the presence of artifacts in the v-HIT trace.

## Conclusion

The v-HIT can help to more efficiently decide which side to implant with minimal risk of inducing bilateral vestibular areflexia. Adding the presence of corrective saccades to the evaluation of the v-HIT gain improves the diagnostic power of the v-HIT to determine which CI-candidates need additional caloric testing to detect nuanced differences in case of a significant vestibular loss, which the v-HIT is unable to predict by itself. The newly developed model MGS balances the sensitivity and specificity of the v-HIT better than the more commonly evaluated parameters such as mean gain, MinGain_LR and GA.

## Data Availability Statement

The raw data supporting the conclusions of this article will be made available by the authors, without undue reservation.

## Ethics Statement

The studies involving human participants were reviewed and approved by LUMC LDD G21.007. Written informed consent for participation was not required for this study in accordance with the national legislation and the institutional requirements.

## Author Contributions

JF was responsible for the research idea. JF, JB, BE, CF, and EH were responsible for the study design. CF, BE, and JB for data extraction and statistical analysis. CF, BE, JB, JF, EH, and PB for draft writing and critical revision. All authors contributed to the article and approved the submitted version.

## Conflict of Interest

The authors declare that the research was conducted in the absence of any commercial or financial relationships that could be construed as a potential conflict of interest.
