# Supplementary material for: Saccades Matter: Reduced Need for Caloric Testing of Cochlear Implant Candidates by Joint Analysis of v-HIT Gain and Corrective Saccades
Source: Front Neurol. 2021 Jun 28;12:676812. doi: 10.3389/fneur.2021.676812 (PMC8273162; doi:10.3389/fneur.2021.676812)
Supplement: Supplementary file 1 [file Table_1.docx]

**Supplementary table**. Overview of caloric testing and v-HIT results per CI-candidate. UW: unilateral weakness, DP: direction preponderance, SPV R: slow phase velocity to the right, SPV L: slow phase velocity to the left. Saccades: 1 (presence), 0 (absence).

| **Subject** | **Caloric test outcome** | | | | **v-HIT** **outcome** | | | |  |  |
| --- | --- | --- | --- | --- | --- | --- | --- | --- | --- | --- |
|  | **UW cal (%)** | **DP**  **(%)** | **SPV R (°/s)** | **SPV L (°/s)** | **Gain**  **R** | **Gain**  **L** | **Asymmetry (%)** | **Saccades** | **MGS** | **Mean gain** |
| 1 | 76,5 | 28,5 | 5,4 | 26,2 | 1 | 0,9 | 10 | 1 | 0,90 | 0,95 |
| 2 | 28,4 | 16,3 | 16,8 | 9,4 | 1,1 | 0,95 | 14 | 0 | 1,00 | 1,03 |
| 3 | 17 | 4,6 | 13,9 | 62,6 | 0,99 | 0,91 | 4 | 1 | 0,91 | 1,07 |
| 4 | 4,1 | 4,1 | 26,6 | 28,9 | 1,02 | 0,94 | 8 | 1 | 0,94 | 0,98 |
| 5 | 25,7 | 25,7 | 13,5 | 22,8 | 0,98 | 0,88 | 10 | 0 | 1 | 0,93 |
| 6 | 30,7 | 6,9 | 27,3 | 14,5 | 1,07 | 0,96 | 10 | 0 | 1 | 1,02 |
| 7 | 3,9 | 7,8 | 52,6 | 57 | 1,03 | 1,06 | 3 | 0 | 1 | 1,05 |
| 8 | 32,60 | 9,1 | 5,7 | 11,2 | 0,75 | 0,72 | 4 | 0 | 0,72 | 0,74 |
| 9 | 0,3 | 11,5 | 70,2 | 70,6 | 0,93 | 0,88 | 5 | 0 | 1 | 0,91 |
| 10 | 54,7 | 23 | 19,7 | 67,1 | 0,77 | 0,85 | 9 | 1 | 0,77 | 0,81 |
| 11 | 40,50 | 85,3 | 5 | 5,8 | 0,85 | 0,74 | 10 | 1 | 0,74 | 0,78 |
| 12 | 100 | 100 | 0 | 0 | 0,95 | 0,76 | 20 | 1 | 0,76 | 0,86 |
| 13 | 32,9 | 16,4 | 25,2 | 49,9 | 0,94 | 0,86 | 9 | 0 | 1 | 0,90 |
| 14 | 40,1 | 28,1 | 23,9 | 55,9 | 1,06 | 0,9 | 15 | 0 | 1 | 0,98 |
| 15 | 22,9 | 20,7 | 41,8 | 26,2 | 0,99 | 0,98 | 1 | 1 | 0,98 | 0,99 |
| 16 | 8,8 | 5,8 | 38,8 | 46,6 | 1,18 | 0,95 | 19 | 1 | 0,95 | 1,07 |
| 17 | 25,6 | 23,4 | 2,3 | 3,9 | 0,99 | 0,77 | 22 | 0 | 0,77 | 0,88 |
| 18 | 79,10 | 14,6 | 70,6 | 8,3 | 1,08 | 0,9 | 17 | 1 | 0,90 | 0,99 |
| 19 | 26,2 | 7,4 | 12,2 | 21 | 0,98 | 0,91 | 7 | 1 | 0,91 | 0,95 |
| 20 | 32,8 | 32,8 | 13,2 | 26,1 | 1,05 | 0,98 | 7 | 0 | 1 | 1,02 |
| 21 | 20,2 | 13,4 | 26,6 | 40 | 0,96 | 0,86 | 10 | 0 | 1 | 0,91 |
| 22 | 13 | 24,9 | 19,6 | 15,1 | 0,99 | 0,88 | 11 | 0 | 1 | 0,94 |
| 23 | 3,2 | 11,1 | 31,7 | 33,9 | 1,01 | 0,93 | 8 | 0 | 1 | 0,97 |
| 24 | 6,2 | 6,2 | 3,9 | 3,5 | 0,05 | 0,17 | 71 | 1 | 0,05 | 0,11 |
| 25 | 6,6 | 14,6 | 48,8 | 55,7 | 0,77 | 0,73 | 5 | 0 | 0,73 | 0,75 |
| 26 | 1,4 | 5,6 | 66,1 | 68 | 1 | 0,92 | 8 | 0 | 1 | 0,96 |
| 27 | 35,30 | 32,8 | 23 | 11 | 0,87 | 0,78 | 10 | 1 | 0,78 | 0,83 |
| 28 | 5,6 | 18,7 | 13,2 | 14,8 | 0,87 | 0,83 | 5 | 1 | 0,83 | 0,85 |
| 29 | 100 | 100 | 1,5 | 2 | 0,05 | 0,11 | 55 | 1 | 0,05 | 0,08 |
| 30 | 6,7 | 6,3 | 21,2 | 24,2 | 0,97 | 0,91 | 6 | 0 | 1 | 0,94 |
| 31 | 21,40 | 5,4 | 14,5 | 9,4 | 1,26 | 1,05 | 17 | 1 | 1,05 | 1,16 |
| 32 | 22,3 | 20,3 | 27,3 | 17,4 | 0,82 | 0,63 | 23 | 1 | 0,63 | 0,73 |
| 33 | 9 | 3 | 16 | 13 | 0,95 | 0,88 | 7 | 0 | 1 | 0,92 |
| 34 | 13 | 17,2 | 123,1 | 94,8 | 1,21 | 0,95 | 21 | 0 | 1 | 1,08 |
| 35 | 100 | 100 | 1,2 | 1,8 | 0,35 | 0,53 | 34 | 1 | 0,35 | 0,44 |
| 36 | 24,50 | 41,1 | 9,7 | 15,9 | 0,99 | 0,9 | 9 | 1 | 0,90 | 0,95 |
| 37 | 7,7 | 0 | 54,5 | 46,8 | 1,18 | 1,15 | 3 | 1 | 1,15 | 1,17 |
| 38 | 11,40 | 11,4 | 3,1 | 2,5 | 0,93 | 0,93 | 0 | 1 | 0,93 | 0,93 |
| 39 | 52,40 | 8 | 7 | 22,6 | 0,92 | 0,79 | 14 | 0 | 0,79 | 0,86 |
| 40 | 62,30 | 62,3 | 36 | 11 | 0,99 | 0,77 | 22 | 1 | 0,77 | 0,88 |
| 41 | 24,8 | 15,9 | 35,6 | 59 | 0,91 | 0,79 | 13 | 0 | 0,79 | 0,85 |
| 42 | 40 | 25 | 20 | 45 | 0,98 | 0,91 | 7 | 1 | 0,91 | 0,95 |
| 43 | 0,2 | 2,5 | 18,8 | 18,8 | 0,88 | 0,92 | 4 | 1 | 0,88 | 0,90 |
| 44 | 2,6 | 9,6 | 85,8 | 81,4 | 1,31 | 1,15 | 12 | 1 | 1,15 | 1,23 |
| 45 | 4,7 | 0,9 | 17,6 | 19,4 | 1 | 0,86 | 14 | 0 | 1 | 0,93 |
| 46 | 6,1 | 9 | 73,7 | 65,2 | 0,99 | 0,96 | 3 | 0 | 1 | 0,98 |
| 47 | 60 | 18,5 | 90 | 22,4 | 1,08 | 1,02 | 6 | 1 | 1,02 | 1,05 |
| 48 | 0,7 | 11,4 | 10,6 | 10,6 | 1,36 | 1,06 | 22 | 1 | 1,06 | 1,21 |
| 49 | 29,3 | 13,8 | 32,9 | 17,9 | 0,94 | 0,87 | 7 | 0 | 1 | 0,91 |
| 50 | 100 | 100 | 0 | 5,2 | 0,47 | 0,67 | 30 | 1 | 0,47 | 0,57 |
| 51 | 35,1 | 8,3 | 37,2 | 77,2 | 0,93 | 0,85 | 9 | 1 | 0,85 | 0,89 |
| 52 | 25,3 | 21,5 | 56,1 | 93,9 | 1,18 | 0,94 | 20 | 1 | 0,94 | 1,06 |
| 53 | 5 | 34,2 | 23,3 | 21,1 | 1,01 | 0,98 | 3 | 1 | 0,98 | 1 |
| 54 | 50,90 | 34,1 | 7,8 | 8,9 | 1,19 | 0,93 | 22 | 1 | 0,93 | 1,06 |
| 55 | 13,7 | 2,8 | 33 | 43,6 | 1,09 | 0,96 | 12 | 1 | 0,96 | 1,03 |
| 56 | 3 | 8,9 | 21,1 | 19,8 | 0,98 | 0,9 | 8 | 0 | 1 | 0,94 |
| 57 | 4,3 | 2,9 | 39,2 | 42,7 | 0,99 | 0,89 | 10 | 0 | 1 | 0,94 |
| 58 | 0,5 | 2,1 | 32,6 | 32,4 | 1,06 | 0,99 | 7 | 0 | 1 | 1,03 |
| 59 | 5,3 | 5,3 | 57,9 | 64,4 | 0,85 | 0,83 | 2 | 1 | 0,83 | 0,84 |
| 60 | 7,7 | 10,5 | 26,4 | 30,8 | 1,13 | 0,9 | 20 | 0 | 1 | 1,02 |
| 61 | 59,60 | 59,6 | 3,9 | 15,6 | 0,6 | 0,91 | 34 | 1 | 0,6 | 0,76 |
| 62 | 1,6 | 12,9 | 35,7 | 34,6 | 1,11 | 1,01 | 9 | 1 | 1,01 | 1,06 |
| 63 | 3,6 | 10,3 | 63,2 | 67,9 | 1,02 | 0,95 | 7 | 0 | 1 | 0,99 |
| 64 | 19,2 | 19,2 | 26,5 | 27 | 0,91 | 0,77 | 15 | 1 | 0,77 | 0,84 |
| 65 | 20,6 | 35,2 | 16,4 | 25 | 0,97 | 0,85 | 12 | 0 | 1 | 0,91 |
| 66 | 35,20 | 5,4 | 22,4 | 10,7 | 0,98 | 1,03 | 5 | 1 | 0,98 | 1,01 |
| 67 | 15,1 | 17,8 | 16 | 21,7 | 1,03 | 1,01 | 2 | 1 | 1,01 | 1,02 |
| 68 | 61,70 | 35,2 | 5,7 | 23,8 | 1,02 | 0,93 | 9 | 1 | 0,93 | 0,98 |
| 69 | 15 | 23 | 22 | 16 | 0,98 | 0,91 | 7 | 1 | 0,91 | 0,95 |
| 70 | 6,3 | 6,3 | 59,8 | 52,7 | 1,13 | 1,05 | 7 | 1 | 1,05 | 1,09 |
| 71 | 32,2 | 23,1 | 29 | 56,5 | 0,63 | 0,63 | 0 | 0 | 0,63 | 0,63 |
| 72 | 20,9 | 16 | 46,4 | 30,4 | 1 | 0,96 | 4 | 1 | 0,96 | 0,98 |
| 73 | 9,10 | 28,6 | 6,3 | 5,2 | 0,84 | 0,8 | 7 | 1 | 0,80 | 0,82 |
| 74 | 27,8 | 4,8 | 37,6 | 66,6 | 1 | 1 | 0 | 1 | 1 | 1 |
| 75 | ,60 | 15 | 26,8 | 26,4 | 0,32 | 0,8 | 60 | 1 | 0,32 | 0,56 |
| 76 | 55,70 | 14,8 | 30 | 8,5 | 0,95 | 0,8 | 16 | 1 | 0,80 | 0,88 |
| 77 | 29,4 | 19,7 | 26,1 | 47,9 | 1,19 | 0,95 | 20 | 0 | 1 | 1,07 |
| 78 | 27,2 | 27,2 | 26 | 39,4 | 0,72 | 0,72 | 0 | 1 | 0,72 | 0,72 |
| 79 | 17,1 | 24,8 | 97,3 | 68,9 | 1,11 | 1,07 | 4 | 0 | 1 | 1,09 |
| 80 | 25,90 | 36,8 | 7,8 | 4,6 | 0,95 | 0,82 | 14 | 1 | 0,82 | 0,89 |
| 81 | 22,7 | 6 | 52 | 32,8 | 0,91 | 0,87 | 4 | 0 | 1 | 0,89 |
| 82 | 30,10 | 8,8 | 13,3 | 24,7 | 1,1 | 0,97 | 12 | 1 | 0,97 | 1,04 |
| 83 | 23,40 | 7,5 | 12,5 | 7,8 | 0,94 | 0,92 | 2 | 0 | 1 | 0,93 |
